# Rising Intracellular Zinc by Membrane Depolarization and Glucose in Insulin-Secreting Clonal HIT-T15 Beta Cells

**DOI:** 10.1155/2012/190309

**Published:** 2012-03-21

**Authors:** Kira G. Slepchenko, Yang V. Li

**Affiliations:** Department of Biomedical Sciences, Ohio University Heritage College of Osteopathic Medicine, 346 Irvine Hall, Athens, OH 45701, USA

## Abstract

Zinc (Zn^2+^) appears to be intimately involved in insulin metabolism since insulin secretion is correlated with zinc secretion in response to glucose stimulation, but little is known about the regulation of zinc homeostasis in pancreatic beta-cells. This study set out to identify the intracellular zinc transient by imaging free cytosolic zinc in HIT-T15 beta-cells with fluorescent zinc indicators. We observed that membrane depolarization by KCl (30–60 mM) was able to induce a rapid increase in cytosolic concentration of zinc. Multiple zinc transients of similar magnitude were elicited during repeated stimulations. The amplitude of zinc responses was not affected by the removal of extracellular calcium or zinc. However, the half-time of the rising slope was significantly slower after removing extracellular zinc with zinc chelator CaEDTA, suggesting that extracellular zinc affect the initial rising phase of zinc response. Glucose (10 mM) induced substantial and progressive increases in intracellular zinc concentration in a similar way as KCl, with variation in the onset and the duration of zinc mobilization. It is known that the depolarization of beta-cell membrane is coupled with the secretion of insulin. Rising intracellular zinc concentration may act as a critical signaling factor in insulin metabolism of pancreatic beta-cells.

## 1. Introduction

Zinc has been known for decades as a critical cofactor for insulin biosynthesis and storage in pancreatic beta-cells (*β*-cells) [1]. Insulin secretion is correlated with zinc release by the beta cells in response to glucose stimulation [2, 3]. In the presence of zinc within the cell, insulin assembles to a hexamer with two zinc ions in the center. This form of insulin is stored and secreted as insulin-zinc crystal. The link between zinc and diabetes has long been proposed [4]. Hyperglycemia appears to interfere with the zinc status of the body as type II diabetes patients display a marked decrease in total plasma zinc and hyperzincuria. However, the relationship between diabetes, insulin and zinc is complex with no clear cause and affects relationships [5].

Zinc is an important structural and functional component in many cellular proteins and enzymes and plays a key role in normal growth and development as well as normal cell functions. Both zinc deficiency and excess of free zinc are toxic to mammalian cells [6–12]. As such, zinc is normally tightly regulated, limiting the extent of cytosolic labile (or free) zinc concentration [13, 14]. The advances in the field of zinc biology over the last decade have facilitated our understanding of zinc trafficking, intracellular distribution, and storage. The intracellular zinc concentration is buffered not only by metalloproteins but also by organelle sequestration [15]. Currently, two categories of zinc transporters have been identified for the cellular zinc trafficking and storage [7]. The level of free zinc appears to mediate multiple signaling pathways including apoptotic signaling cascades [16–19], indicating that zinc can act as an intracellular signaling molecule and regulate cellular metabolism and functional activities.

Although zinc appears to be intimately involved in insulin metabolism, little is known about the regulation of zinc homeostasis in pancreatic beta cells [20, 21]. Studies based on analysis of islets from ^65^Zn-treated rats have shown that the secretory granules contain one-third of the total beta-cell zinc with zinc concentrations reaching 10–20 mM in the interior of the dense-core granule [3, 22]. Diminishing granule zinc with various chelators has induced diabetes in some animal models [23]. It is presumed that there is an active zinc trafficking mechanism to maintain adequate amounts of zinc in beta-cells. We hypothesize that there may be a zinc transient or changes in cytosolic zinc during glucose application, which could be essential for the proper function of pancreatic beta-cells. The present study set out to investigate the intracellular zinc transient by imaging free cytosolic zinc in HIT-T15 beta-cell line with fluorescent zinc indicators. The results show that membrane depolarization induced a rapid and substantial increase in cytosolic zinc concentration. Glucose also induced an increase in cytosolic zinc. Since depolarization of beta-cell membrane is coupled with the secretion of insulin, rising zinc may act as a signaling factor in insulin metabolism of pancreatic beta-cells.

## 2. Materials and Methods

### 2.1. Cell Culture

 Clonal HIT-T15 cells were gift-from Dr. Calvin James (Ohio University) ATTC (number CRL-1777). Cells were used between passages 61–78. In the constraint of these passages, cells maintained normal insulin secretion and glucose sensitivity. They were maintained in Ham's F12K medium supplemented with 2.5% fetal bovine serum and 10% dialyzed horse serum in 5% CO_2_–95% humidified air at 37°C (as suggested by ATCC). Cells were passaged once a week (confluency about 70%) using 0.25% trypsin-EDTA. 

### 2.2. Fluorescent Experiments

For fluorescent experiments, 70% confluent HIT-T15 cells were detached with trypsin and plated into 35 mm glass bottom Petri dishes (MatTek Corp. P35G-4.5-14-C) with 2 mL of Ham's F12K medium supplemented with 2.5% fetal bovine serum and 10% dialyzed horse serum and were maintained in 5% CO_2_–95% humidified air at 37°C from 48 to 72 hours prior to the experiments. Two- to three-day old HIT-T15 cells plated into 35 mm glass bottom dishes were washed with 1 mL of basal HEPES buffer (each concentration is in mM) 25 HEPES, 125 NaCl, 3 KCl, 1.28 CaCl_2_, 1.1 MgCl_2_, 0.8 glucose, and pH 7.4. Washing was repeated two more times, and cells were loaded in 5% CO_2_–95% humidified air at 37°C with 5 *μ*M FluoZin-3 AM or 5 *μ*M Newport Green DCF diacetate. Each loading was done for 30 minutes. After loading was complete, cells were washed with 1 mL buffer two times and left to rest in 1 mL of basal buffer at room temperature for 10 min prior to data collection. Images of cells were taken using camera QImaging Retiga 1300i on inverted microscope MoticAE31 using Olympus U Plan FL 40X, 0.75 NA. All experiments were done at room temperature.

For potassium chloride (KCl) stimulation, the stimulating medium consisted of (each concentration is in mM) 25 HEPES, 120 mM KCl, 5 NaCl, 1.28 CaCl_2_, 1.1 MgCl_2_, 0.8 mM glucose, and pH 7.4. The final KCl concentration during the stimulation experiments was 60 mM, unless otherwise specified. Images of cells were acquired every 1 or 5 sec with exposure time 300–700 ms. For glucose stimulation, the stimulating medium consisted of (each concentration is in mM) 25 HEPES, 125 NaCl, 3 KCl, 1.28 CaCl_2_, 1.1 MgCl_2_, 20 glucose, and pH 7.4. Final glucose concentration was 10 mM. These stimulation mediums were prepared and stored in double concentration stocks. Cells were bathed in basal buffer (0.8 mM glucose) for 30 min in 5% CO_2_–95% humidified air at 37°C, before loading with fluorescent indicators in same buffer in 5% CO_2_–95% humidified air at 37°C. Cells were then rinsed in basal buffer and incubated in it for 10 min at room temperature before each experiment. Care was taken to minimize potential zinc contamination that might bring in background fluorescence. Serum and any other source of proteins were not used in buffers made for experiments. Every reasonable precaution was taken not to contaminate our experimental medium/buffer with zinc. Purest water was used to prepare buffers (Ultra Pure Polishing System, USFilter); only Teflon plastic containers were used to make and store buffers, to minimize zinc leakage into buffers from the glassware. The testing buffer/solution was made with puriss grade salts (Sigma or Fluka), and chemicals were transferred with Spatulas coated with tygon or polymethylpentene (Nalgene).

### 2.3. Data Analysis

 Fluorescent images were collected and analyzed using Image-Pro 6.2 software (Media Cybernetics). Multiple regions of each cell were highlighted for analysis and tracks generated by Image-Pro software were exported into Excel spreadsheet, where individual tracks were graphed and labeled. Data is presented as moving average of 2–4. All possible measures were taken to analyze individual cell; however, some presented data was collected from cells clusters.

### 2.4. Chemicals and Reagents

 Cells culture medium F-12 K (Kaighn's modification of Ham's F-12, with L-glutamine) and fetal bovine serum were purchased from ATCC (catalogue numbers 30—2004 and 30—2021). Trypsin-EDTA 0.25% was obtained from Gibco (catalogue number 25200). Dialyzed Horse Serum (DH-09) was purchased from Omega Scientific. Fluorescent zinc indicators FluoZin-3 (F24195) and Newport Green AM (N7991) as well as pluronic acid were purchased from Invitrogen. Dimethyl sulfoxide and most of the other chemical used in buffers preparation were purchased from Sigma.

## 3. Results

**Figure 1:**
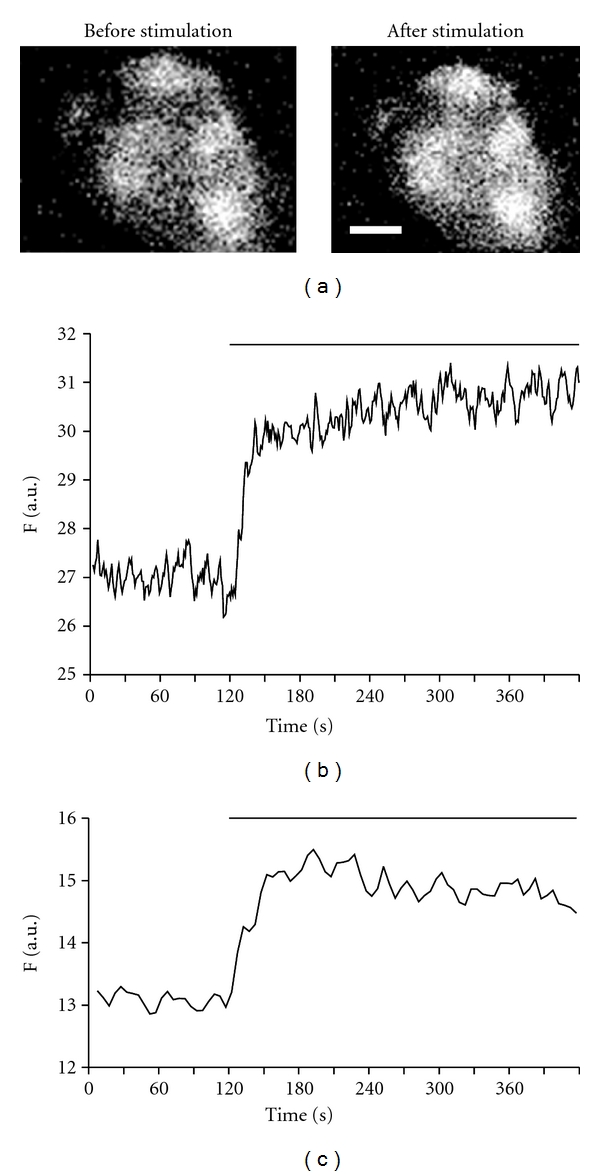
Depolarization induced intracellular zinc rise in HIT-T15 beta-cells. (a) Images of beta-cells loaded with 5 *μ*M FluoZin-3 AM before and after 60 mM KCl application. Scale bars are 10 *μ*m. (b) Representative trace showing KCl (60 mM) induced intracellular zinc rise in cells loaded with 5 *μ*M FluoZin-3. Line above the trace indicates KCl application. (c) Representative trace showing KCl (60 mM) induced intracellular zinc rise in cells loaded with 5 *μ*M Newport Green.

Beta cells loaded with fluorescent zinc indicator FluoZin-3 AM (*K*_*D*  zinc_ ≈ 15 nM, 5 *μ*M) responded with an increase in intracellular zinc upon depolarization with KCl, a well-established means of depolarizing membrane to cause release of insulin from pancreatic beta-cells. The kinetics of the intracellular zinc rise was transient. As shown in Figure 1, the onset of rising intracellular zinc was considerably quick and could be detected within a few seconds after the application of KCl. The intracellular zinc increased to its peak within about one min. This initial fast rising of KCl-induced zinc transient lasted about 10–20 sec, but reached more than 70% of peak response. After reaching the peak, the KCl induced-zinc response did not return to baseline due to the continuous presence of KCl during entire time (3–5 min) of the recording. There were clusters of brighter zinc fluorescence in each FluoZin-3 loaded beta-cell, which appeared to be distributed in secretory vesicles or granules, similar to zinc distribution observed in FluoZin-3-loaded MIN6 cells [24]. The high KCl-evoked zinc rises were observed in all analyzed cells. The increase in intracellular zinc by membrane depolarization was also verified with another fluorescent zinc indicator Newport Green (*K*_*D*  zinc_≈ 1–3 *μ*M, 5 *μ*M). The effect of treatment with KCl in Newport Green loaded beta-cells was similar to those obtained in FluoZin-3-loaded cells. However, the zinc response declined earlier, probably due to a relatively low affinity of Newport Green indicator. These results indicated that the application of KCl induced a substantial zinc increase in HIT-T15 beta-cells.

**Figure 2:**
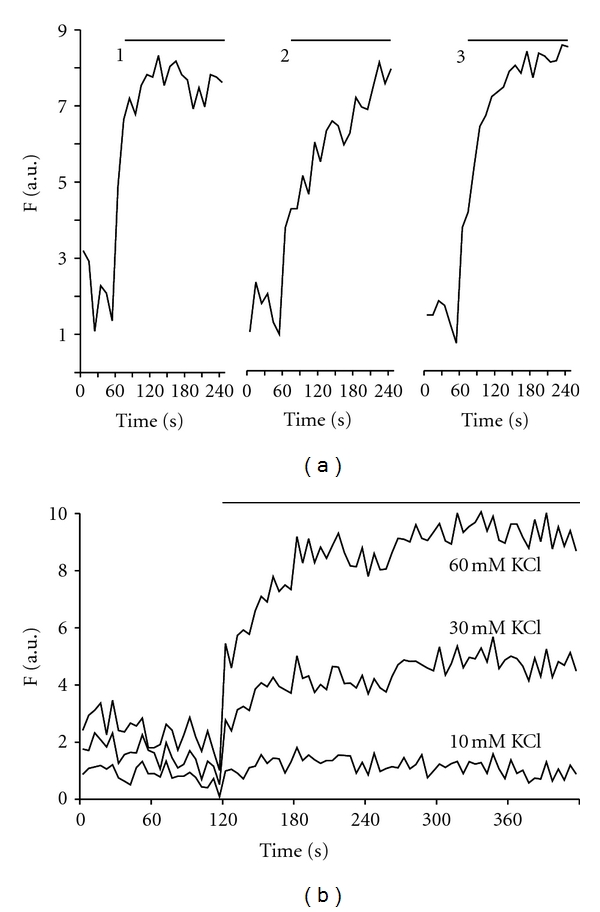
Zinc transients induced by repeated KCl applications and concentration dependence. (a) Repeatable responses to three consecutive KCl (60 mM) applications in the same cells. There were 10 min intervals between stimulations. Two experiments were done with about 10 cells per experiment. (b) The intracellular zinc rises were induced by 10, 30, and 60 mM KCl applications, respectively. Experiments were done in the same cells. Each presented trace is representative of at least two different experiments.

We studied beta-cells zinc responses to repeated potassium stimulations. After response to the first stimulation, cells were washed once with basal buffer and left to rest for 10 min. The second KCl stimulation and data collection were done using the same parameters as the first application; same steps were repeated to observe the third KCl-induced response. The multiple application of KCl elicited repeated intracellular zinc increases. The amplitude of the response with repeated KCl stimulation was generally similar to each other (Figure 2(a)). We also found that the KCl induced intracellular zinc increase in beta-cells was concentration-dependent and could be detected when beta-cells were stimulated with as little as 10 mM KCl application. The application of 30 mM KCl induced steady intracellular zinc increases. These results suggest that intracellular zinc increases in beta-cells varied continuously with the degree of stimulation.

**Figure 3:**
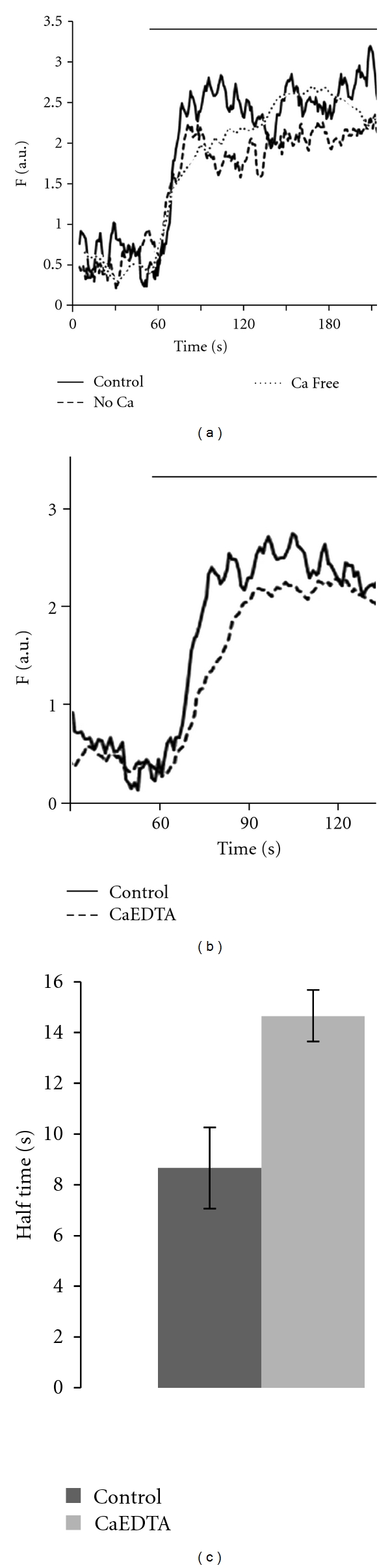
Effect of extracellular calcium or zinc on KCl induced intracellular zinc rise. (a) KCl (60 mM) induced intracellular zinc rises in the presence or absence of extracellular calcium. The latter was achieved by bathing cells with “no calcium” medium (no calcium addition) or “calcium-free medium” (no calcium addition plus 500 *μ*M EGTA). (b) KCl (60 mM) induced intracellular zinc rise in the control medium and the medium containing 1 mM CaEDTA to remove extracellular zinc. In (a) and (b), each experiment was done on separate set of cells. (c) Comparison of half time in seconds to reach the maximum stimulation induced by KCl alone (*N* = 10) and KCl with 1 mM CaEDTA (*N* = 9), *P* < 0.01. Each *point* represents the averaged, normalized zinc rise initial slope ± SEM from data in (b).

Additional tests were undertaken to determine whether the depolarization-induced intracellular zinc rise was affected by the status of extracellular calcium or zinc. As shown in Figure 3, the intracellular zinc rise was generally not dependent on the presence of either extracellular calcium or extracellular zinc. Omission of calcium from medium bathing beta-cells did not reduce the intracellular zinc rise induced by the application of high KCl (Figure 3(a)). The removal of extracellular zinc by chelating zinc with CaEDTA (1 mM), an extracellular zinc chelator, did not reduce zinc rise either; however, close-up analysis revealed that removing the extracellular zinc caused a slower KCl-induced intracellular zinc rise as comparing to the control.

**Figure 4:**
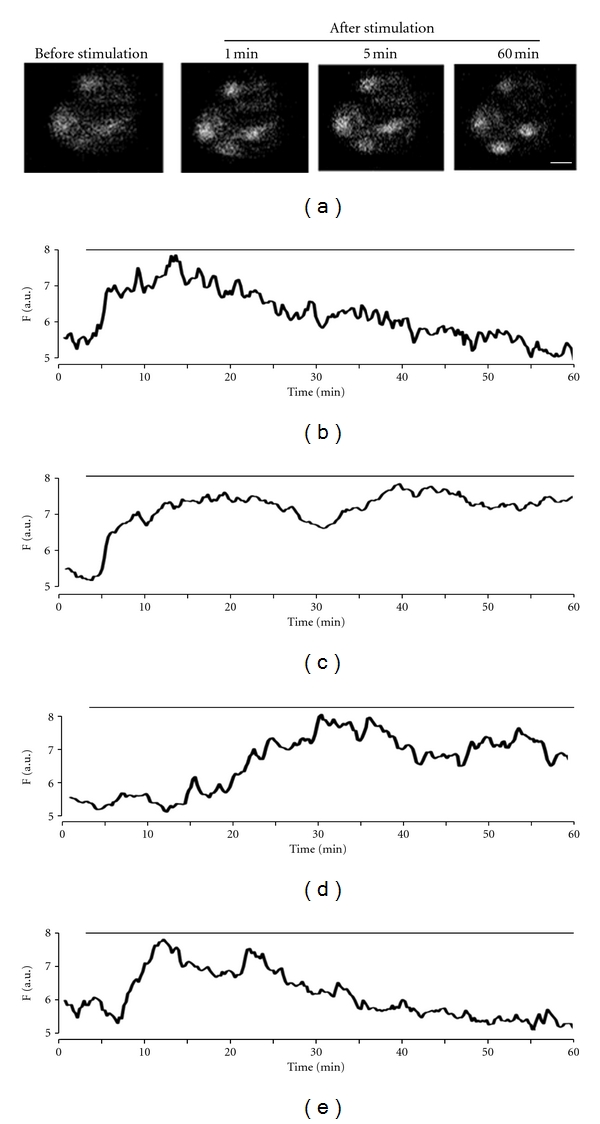
Intracellular zinc responses to glucose stimulation in HIT-T15 beta-cells. (a) Images of beta-cells loaded with 5 *μ*M FluoZin-3 AM before and after 10 mM glucose application. Scale bars are 10 *μ*m. (b)–(e) Heterogeneous increases of intracellular zinc in the presence of stimulating glucose (10 mM): early onset response ((b); **N** = 8), late onset response ((d); **N** = 2), oscillation- or wave-like responses ((c), (e); **N** = 3). The line above traces indicates the continuous presence of 10 mM glucose.

Glucose induced the intracellular rises in HIT-T15 beta-cells, in a similar manner as KCl. The cells were bathed in medium containing 0.8 mM glucose for about 60 min before 10 mM glucose stimulation. As shown in Figure 4, the zinc responses to glucose stimulation (10 mM) were heterogeneous in the onset and the duration of their zinc mobilization. The most cells exhibited an early onset response with zinc response declined progressively in the continuous presence of glucose (Figure 4(b)). More complex zinc responses (e.g., oscillation- or wave-like responses) were also observed (Figures 4(c), 4(d), and 4(e)).

## 4. Discussion

The main finding in the present study is that membrane depolarization with high-concentration KCl is able to induce a steady increase in intracellular concentration of zinc. Multiple similar zinc transients were elicited during repeated stimulation with KCl. The amplitude of zinc transients was not affected by the absence of extracellular calcium or extracellular zinc. However, the half time of the rising slope was slower after removing extracellular zinc, suggesting that extracellular zinc appeared to affect or contribute to kinetics of the initial rising phase of zinc response. Glucose treatment also induced an increase in intracellular zinc. There was a substantial and progressive rise in response to glucose stimulation. Since depolarization of beta-cell membrane is coupled with the secretion of insulin, rising intracellular zinc may act as a critical signaling factor in insulin metabolism of pancreatic beta-cell.

Available data supports that intracellular zinc levels can be determined by the interaction of membrane zinc transporters and cytoplasmic zinc buffers [7, 25]. The total zinc content of the mammalian pancreas is significantly higher in beta-cells than in other cell types [26], with zinc concentration reaching 10–20 mM in the interior of the dense-core granules [3, 22]. Similar to other cell types [27, 28], free cytosolic zinc concentration in beta-cells appear to be tightly regulated to maintain cytosolic zinc concentration in a rather low range about ~400 pM [20]. In the present study, the increase in intracellular zinc by KCl was not only detected with fluorescent zinc indicator FluoZin-3 (*K*_*D*  zinc_ ≈ 15 nM) but also verified with the low-affinity zinc indicator Newport Green (*K*_*D*  zinc_ ≈ 1–3 *μ*M), suggesting a substantial increase in intracellular zinc concentration. Based on the affinity of fluorescent indicators to zinc and the range of its fluorescence detection previously described [29], the peak concentration of the zinc transient was in the range of high nM and low *μ*M. Since these acetoxymethyl or acetate ester-derivatized indicators were hydrolyzed by ubiquitous esterases in the cytosolic membrane [30] before they could penetrate granules, they most likely measured free or labile zinc in cytosolic space. The above findings are in line with an earlier report by Bellomo et al. [20], who observed an increase in intracellular free zinc in beta-cells in response to prolonged glucose stimulation (2 or 24 hr) using a genetically engineered (FRET)-based sensor (eCALWY-4).

The glucose stimulation in beta-cells produced a response of heterogeneous zinc response. Since zinc is actively involved in insulin metabolism, it seems reasonable to speculate that the change in cytosolic zinc by glucose will potentially affect secretion or availability of insulin in beta-cells. Our study shows that KCl-induced intracellular zinc transients are largely independent of extracellular zinc, indicating that the observed increases in cytosolic zinc may result primarily from intracellular compartments. However, the removal of extracellular zinc by chelator CaEDTA reduced the slope of KCl-induced zinc increases. The latter implies that the entry of extracellular zinc may contribute to the early phase of zinc transient. Calcium-independent zinc rise as shown in the present study suggests that the rising zinc does not affect insulin-secretion process, as calcium influx is required for the sustained secretion of insulin. Extracellular calcium-independence of KCl-induced intracellular calcium transient and substance releases (peptides or neurotransmitters) have been reported [31–34] and were shown to involve phospholipase C_*β*_ (PLC_*β*_) pathways coupled with voltage sensitive receptors [31]. Similar results were reported in beta-cells, showing KCl or glucose induced intracellular calcium transient was dependent upon depolarization alone, possibly through increasing inositol 1,4,5-trisphosphate production [35], also see [36, 37]. Whether the similar mechanism is also involved in stimulation-induced zinc increases in beta-cells needs further study.

The present study shows robust zinc fluorescence in response to multiple stimulations, suggesting a rapid equilibrium in the zinc compartment measured. This result supports the possible existence of an active mechanism that regulates cytosolic zinc trafficking within the pancreatic beta-cells. The rapid equilibrium between different compartments such as cytosolic and extracellular zinc has long been reported [3, 22]. Recent studies indicate that zinc homoeostasis is regulated by two large metal-transporter families: the Zip family that mediates zinc influx into the cytosol and the ZnT family that facilitates zinc efflux or clearance from the cytosol into intracellular cell compartments or out of the cell [38]. While the uptake (or reuptake) of zinc ions is likely to be required to maintain an adequate intracellular zinc level, various mechanisms have been proposed to explain intracellular zinc fluctuations at the single cell level, including metallothioneins and storages in endoplasmic reticulum (ER) and mitochondria [39, 40]. For example, ZnT5/ZnT6 hetero-oligomeric complexes are involved in ER homeostasis by transporting zinc under stress conditions [38, 41] and may function as bidirectional transporters [7, 42–44]. ZIP7 and ZIP8 appear to be expressed in the plasma membrane of Golgi apparatus and lysosomes respectively [45, 46]. A number of zinc transporters have been found to be expressed in beta-cells including ZnT1, ZnT4, ZnT5, ZnT6, ZnT7, and ZnT-8 of the SLC30A family, and ZIP1, ZIP10, and ZIP14 of the SLC39A family [47]. Among them, ZnT8 is a granule-specific zinc transporter. It has been recently shown that mice lacking ZnT8 have reduced intragranular zinc [48]. The latter is associated with an increased risk of type 2 diabetes [49–52].

The present study suggests that glucose may prompt the cytosolic zinc trafficking within the pancreatic beta-cell. What is the functional implication of glucose-induced zinc transients in pancreatic beta-cells? It could simply be the mechanism of refilling granules after insulin/zinc secretion. It could also be a mechanism that regulates insulin metabolism or secretion. The zinc transient could be a critical signal required for the enhanced synthesis or storage of insulin. There is little disputing that zinc is involved in beta-cell insulin metabolism; however, whether it regulates insulin metabolism needs to be further studied.
